# β‐Blockade attenuates renal blood flow in experimental endotoxic shock by reducing perfusion pressure

**DOI:** 10.14814/phy2.14301

**Published:** 2019-12-09

**Authors:** Lex M. van Loon, Gerard A. Rongen, Johannes G. van der Hoeven, Peter H. Veltink, Joris Lemson

**Affiliations:** ^1^ Cardiovascular and Respiratory Physiology Group Faculty of Science and Technology University of Twente Enschede The Netherlands; ^2^ Department of Intensive Care Medicine Radboud University Medical Center Radboud Institute for Health Sciences Nijmegen The Netherlands; ^3^ Department of Pharmacology and Toxicology Radboud University Medical Center Nijmegen The Netherlands; ^4^ Radboud Center for Infectious diseases Nijmegen The Netherlands; ^5^ Biomedical Signals and Systems Faculty of Electrical Engineering, Mathematics and Computer Science Technical Medical Centre University of Twente Enschede The Netherlands

**Keywords:** acute kidney injury, beta‐blocker, renal autoregulation, renal blood flow, sepsis

## Abstract

Clinical data suggests that heart rate (HR) control with selective β1‐blockers may improve cardiac function during septic shock. However, it seems counterintuitive to start β‐blocker infusion in a shock state when organ blood flow is already low or insufficient. Therefore, we studied the effects of HR control with esmolol, an ultrashort‐ acting β1‐selective adrenoceptor antagonist, on renal blood flow (RBF) and renal autoregulation during early septic shock. In 10 healthy sheep, sepsis was induced by continuous i.v. administration of lipopolysaccharide, while maintained under anesthesia and mechanically ventilated. After successful resuscitation of the septic shock with fluids and vasoactive drugs, esmolol was infused to reduce HR with 30% and was stopped 30‐min after reaching this target. Arterial and venous pressures, and RBF were recorded continuously. Renal autoregulation was evaluated by the response in RBF to renal perfusion pressure (RPP) in both the time domain and frequency domain. During septic shock, β‐blockade with esmolol significantly increased the pressure dependency of RBF to RPP. Stopping esmolol showed the reversibility of the impaired renal autoregulation. Showing that clinical diligence and caution are necessary when treating septic shock with esmolol in the acute phase since esmolol reduced RPP to critical values thereby significantly reducing RBF.

## Graphical abstract

In an acute endotoxic septic shock sheep model, we studied the effects of esmolol (an ultrashort acting ß1‐selective adrenoceptor antagonist) administration on renal blood flow and the static and dynamic renal autoregulation during acute septic shock. Our data reveal that both the resuscitated endotoxin shock and the β‐blocker infusion left the renal autoregulation parameters unchanged. However, esmolol reduced the perfusion pressure to critical values thereby significantly reducing renal blood flow.

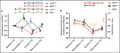

## 1 INTRODUCTION

In critically ill patients, the mortality rate associated with acute kidney injury (AKI) remains high (Brivet, Kleinknecht, Loirat, & Landais, 1996; Jörres, 2002; Nash, Hafeez, & Hou, 2002; Thadhani, Pascual, & Bonventre, 1996). Sepsis and, in particular, septic shock is an important risk factor for developing AKI (Brivet et al., 1996; Jörres, 2002). There is evidence in septic shock that reducing sympathetic outflow, or blocking the action of catecholamines by administering esmolol (an ultrashort acting β1‐selective adrenoceptor antagonist), may improve survival (Morelli et al., 2013). This survival benefit is attributed to improved cardiac function with lower oxygen consumption (Sanfilippo, Santonocito, Morelli, & Foex, 2015). However, it is not clear how organ perfusion, and the perfusion of the kidneys in particular, are affected by β‐blocker infusion during septic shock. It seems counterintuitive to start β‐blocker infusion in a shock state when organ blood flow is already low or insufficient as β‐blockers are reported to reduce tissue perfusion pressure by increasing central venous pressure (CVP) (Loon, Hoeven, Veltink, & Lemson, 2018) and/or by decreasing mean arterial pressure (MAP) (Calzavacca et al., 2014; Hosokawa et al., 2017; Kurita, Kawashima, Morita, & Nakajima, 2017; Mathieu et al., 2018; Suzuki et al., 2005). Besides RPP, the influence of β‐blockers on RBF is further complicated by the unknown effect on renal autoregulation in sepsis or septic shock. Renal autoregulation is mediated by vascular reactivity, unlinking RPP from RBF (Post and Vincent, (2018)). In health, renal autoregulation keeps blood flow at a constant level at perfusion pressures greater than approximately 60–100 mmHg, depending on species (Post, Kellum, Bellomo, & Vincent, 2017). Impaired renal autoregulation will expose the kidney to rapid alterations in blood pressure, resulting in hypotensive or hypertensive injury (Bidani & Griffin, 2004).

Assessment and interpretation of renal autoregulation is not trivial (Cupples & Braam, 2006; Loutzenhiser, Griffin, Williamson, & Bidani, 2006). Studies have addressed either static or dynamic autoregulation: static refers to RPP and RBF values under steady‐state conditions that are observed over a time scale of minutes to hours, while dynamic refers to transient RPP and RBF changes that are observed in a time scale of seconds (Fantini, Sassaroli, Tgavalekos, & Kornbluth, 2016). Even though the mechanisms underlying static and dynamic renal autoregulation may overlap, lack of correlations between the two emphasizes the need to assess both (Jong, Tarumi, Liu, Zhang, & Claassen, 2017).

In this work, we evaluated the effects of esmolol administration on RBF and the static and dynamic renal autoregulation in an experimental animal model of acute septic shock.

## 2 MATERIALS AND METHODS

### 2.1 General

This experiment was performed after approval by the local ethics committee on animal research of the Radboud University Nijmegen Medical Center (RUNMC License number RU‐DEC 2014–10) and in full compliance with Dutch and European legal requirements on the use and protection of laboratory animals. Ten conventionally reared female lambs (crossbred Texelaar‐Flevolanders) were studied under general anesthesia. In the context of the principles of replacement, reduction, and refinement for the use of animal models, no control group was considered necessary to answer our research questions.

The reported results are part of a larger experiment in which we studied the influence of esmolol in experimental endotoxic shock. For a detailed description on the anaesthesia, ventilation, surgical preparation we refer to the materials and methods section of our previous publication (Loon et al., 2018), in short.

### 2.2 Experimental model

#### 2.2.1 Anesthesia and ventilation

Premedication consisted of midazolam and ketamine i.m., anesthesia was induced with i.v. administration of propofol. After endotracheal intubation, general anesthesia was maintained using inhalation of isoflurane, the continuous IV administration of sufentanil and rocuronium.

#### 2.2.2 Surgical preparation

All lambs were positioned in dorsal position for inserting the intravascular catheters using surgical cut down procedures, into the right femoral artery and the left internal jugular vein. An ultrasound transit time flow probe (4 mm) (PAX series, Transonic Systems) was placed around the left renal artery after laparotomy for RBF measurement. An ultrasound transit time perivascular flow probe (14 or 16 mm) (PAX series, Transonic Systems) was placed around the main pulmonary artery to measure CO after left thoracotomy for assessing fluid responsiveness during the resuscitation.

#### 2.2.3 Resuscitation of endotoxic shock

After instrumentation and closing all incisions, a stabilization period of 30 min was followed by continuous i.v. administration of lipopolysaccharide (3 μg kg^−1^ hr^−1^) (LPS, US Standard Reference Endotoxin *Escherichia coli* O:113) after a loading dose of 3 μg/kg in order to create a state of endotoxic shock. Only after LPS had induced a 50% reduction in cardiac output or a 25% reduction in ABP, resuscitation was started according to standard clinical protocol (Rhodes et al., 2017). Resuscitation maneuvers consisted of fluid therapy guided by continuous CO measurement and nor‐epinephrine. Dobutamine was administered in case of fluid refractory shock. Dosages and timings are detailed in our previous work (Loon et al., 2018).

#### 2.2.4 Experimental protocol

Thirty minutes after creating a situation of resuscitated endotoxic shock with blood pressure and CO equal to baseline, esmolol (Baxter) was administrated to reduce the HR by 30%. This targeted HR reduction was based on Morelli et al. who reported a similar level of HR reduction by esmolol in patient with septic shock (i.e., from 115 to 85 bpm) (Morelli et al., 2013). Except for dobutamine, resuscitation maneuvers were maintained or increased in order to maintain ABP and cardiac output at baseline values as far as possible. Thirty minutes after reaching the targeted 30% reduction in HR, the esmolol infusion was stopped.

Four phases in our experiment were studied: T0: Baseline (30 min after instrumentation and prior to LPS infusion); T1: Resuscitation (30 min after successfully restoring MAP and cardiac output to baseline values and prior to esmolol infusion); T2: Esmolol (30 min after esmolol infusion); and T3: Stop esmolol (30 min after stopping esmolol and prior to euthanasia).

### 2.3 Data recording

Arterial blood pressure (ABP) and CVP were recorded using a 18G × 10 cm single‐lumen catheter (Leaderflex, Vygon India Pvt Ltd) in the right femoral artery and a 7.5F × 20 cm 3‐lumen central venous catheter which was placed in the left internal jugular vein (*Multicath*, Vygon India Pvt Ltd), respectively. RPP was calculated by subtracting CVP from ABP.

Hemodynamic pressures and flow signals were continuously recorded on a laptop, computer, and stored on a hard disk with a sample rate of 200 Hz by an A/D converter (NI USB‐6211, National Instrument, Austin). Custom‐written MATLAB scripts (Matlab R2017b, The MathWorks Inc. Massachusetts, USA) were used to generate MAP, mean central venous pressure, and mean RBF by low‐pass filtering of the recorded signals (cutoff frequency of 0.5 Hz (Steptoe & Rüddel, 1985), third‐order Butterworth filter applied in the forward and reverse direction for a zero‐phase response). Renal vascular resistance (RVR) was calculated according to Ohm's law, that is, RPP divided by RBF.

### 2.4 Autoregulation

#### 2.4.1 Static time domain

Renal autoregulation in the time domain was analyzed by using a moving correlation between slow changes of flow and pressure in the kidney, called the renovascular reactivity index (RVx) (Rhee et al., 2012). This assessment was performed per study phase by resampling the ABP and RBF waveforms as nonoverlapping 10‐s mean values. The RVx was calculated by performing a continuous moving Pearson correlation between RPP and RBF. The consecutive paired 10‐s averaged RPP and RBF values from 300‐s analysis periods generated 30 data points for inclusion in each Pearson coefficient used to determine the indices. Positive values of RVx indicate that RBF passively depends on RPP (i.e., impaired autoregulation), and negative values indicate autoregulatory reactivity (Rhee et al., 2012).

#### 2.4.2 Static ‐ autoregulation curves

Renal autoregulation curves were constructed by plotting RPP versus the simultaneously measured RBF, and RPP versus simultaneously calculated RVx. RPP and RBF measurements were first normalized to a percentage of their baseline, which was determined as their mean over a 5‐min period prior to LPS infusion. Twenty‐second mean samples of these normalized RBF recordings and RVx values were plotted against their corresponding RPP value. To estimate the lower limit of autoregulation, we used the method developed by Turkstra et al. (Turkstra, Braam, & Koomans, 2000). In short, the data of the autoregulation curves were subjected to nonlinear regression analysis using a sigmoid function. The lower limit of autoregulation was calculated from the RPP versus RBF curve and was then defined as the perfusion pressure, where the third derivative of the fitted curve was 0, which mathematically defines the shoulder in a sigmoidal curve.

#### 2.4.3 Dynamic frequency domain

Transfer function analysis (TFA) was used to assess renal autoregulation in the frequency domain by studying oscillations from the unfiltered pressure to flow signal. These oscillations were visualized in a power spectrum after the time series has been mathematically translated into the frequency domain. The transfer function H(f) between the two signals was calculated according to Equation 1:

(1) $$H\left(f\right)=S_{\text{xy}}\left(f\right)/S_{\text{xx}}\left(f\right)$$

where S_xx_(f) is the autospectrum of changes in RPP and S_xy_(f) is the cross‐spectrum between the input signal (i.e., RPP) and output signal (i.e., RBF). Using this transfer function (Equation 1), the effect of renal autoregulation can be analyzed at a specific frequency of interest (Scully, Mitrou, Braam, Cupples, & Chon, 2013), that is:

(a) Tubuloglomerular feedback (TGF), the low‐frequency (LF) band [0.02–0.05 Hz].
(b) Myogenic response (MR), the high‐frequency (HF) band [0.1–0.3 Hz].
(c) Baroreflex component (BRC), the very‐high‐frequency (VHF) band [0.35–0.7 Hz].

For each of these frequency bands of interest, the gain, phase, and coherence were calculated. The transfer function gain and phase were derived from the real part of H(f) and the imaginary part of H(f), respectively. The gain quantifies to which extend a change in RBF is caused by a change in RPP. A low transfer gain value implies that oscillations in RPP do not translate into flow fluctuations of similar frequency, that is the kidney is effectively autoregulating in the given frequency band (Post & Vincent, 2018). The phase indicates the latency between the RBF and RPP signal. Last, the coherence is a measure of linearity between two signals in a specific frequency range and was acquired by calculating the squared coherence according to Equation 2:

(2) $$\text{MSC}\left(f\right)=S_{\text{xy}}\left(f\right)/\left({\left(S_{\text{xx}}\left(f\right)*S_{\text{yy}}\left(f\right)\right)}^{.5}\right)$$

Coherence approaching 1 suggest a linear relationship, while coherence approaching 0 suggest no relationship between the signals, severe extraneous noise, or a nonlinear relationship. To ensure reliable TFA outcomes, a cutoff value of 0.5 for coherence was used (Meel‐van den Abeelen, Beek, Slump, Panerai, & Claassen, 2014).

### 2.5 Statistical analysis

Prism Statistical Software was used for statistical analysis (Graph‐Pad Prism 5, GraphPad Software Inc.). Normality was assessed using Shapiro–Wilk tests. RPP and RBF values were normalized to baseline. Repeated measures one‐way analysis of variance (ANOVA) and the Bonferroni test were used for multiple post hoc comparisons of the different time points. Differences in relationship between RPP and RBF or RVx were tested using repeated measures two‐way ANOVA (interaction term) between baseline and the other experimental phases (T0 vs. T1, T0 vs. T2, and T0 vs. T3) and between esmolol and the previous and prior state (T1 vs. T2 and T2 vs. T3). A two‐sided *p* < .05 was considered statistically significant.

## 3 RESULTS

### 3.1 General

**Figure 1:**
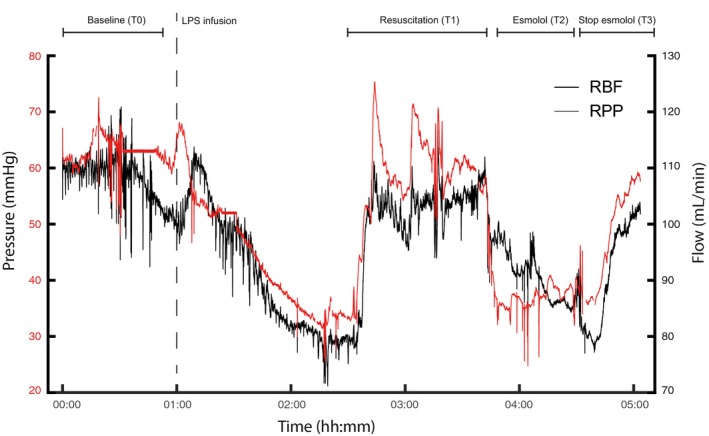
Example of time series renal perfusion pressure (RPP) and renal blood flow (RBF) from lamb 4. Study phases are indicated on top, dashed line marks the start of LPS infusion

**Figure 2:**
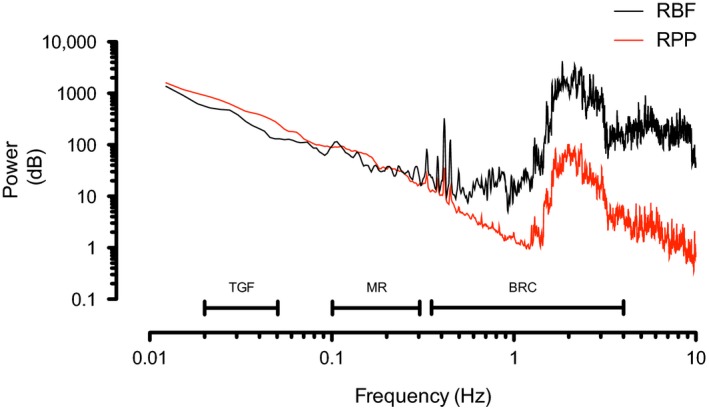
Example of power spectrum of the RPP and RBF signal from lamb 4 during baseline, with renal autoregulatory operating ranges. TGF, Tubuloglomerular feedback, MR, myogenic response, and BRC, Baroreceptor component

**Figure 3:**
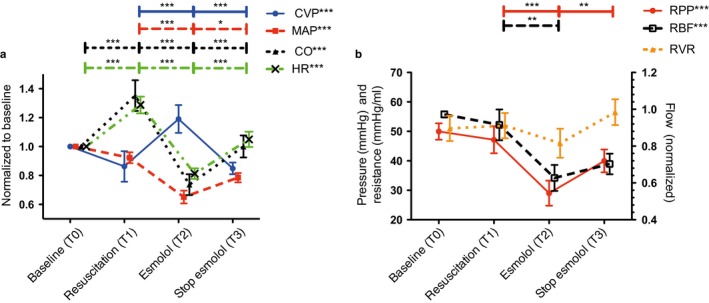
(a) Central venous pressure (CVP), mean arterial pressure (MAP), cardiac output (CO), and heart rate (HR) per study phase. (b) Renal perfusion pressure (RPP), renal blood flow (RBF), and renal vascular resistance (RVR) per study phase. Data are expressed as mean ± *SEM*. Repeated measures ANOVA was performed for all three parameters. Paired Student's *t* test was used to perform pairwise comparisons between phases. *p < .05, **p < .01, and ***p < .001

A total of 10 ewe lambs (age 6–8 months, mean weight of 20.9 kg [range: 13–24.5 kg], mean body surface area of 0.94 m^2^ [range: 0.67–1.0 m^2^] were studied; two lambs were excluded because of insufficient quality of RBF recordings. All animals included in the study were considered healthy on physical examination when entering the animals’ laboratory. The resuscitation maneuvers in combination with the continues LPS infusion resulted in endotoxic shock symptoms, with increased CO, tachycardia, and reduced MAP (Figures 1, 2 and 3a). Concurrent esmolol infusion induced on average a HR reduction of 37% [range: 31%–41%] (Figures 1, 2 and 3a).

### 3.2 Pressure, flow, and resistance

The median RPP at baseline was 48 mmHg [range: 20–55 mmHg]. Resuscitation maneuvers were able to maintain RPP after LPS infusion, while the esmolol infusion decreased RPP (T2) (Table 1). The RPP recovered after discontinuing the esmolol infusion. RBF followed the same pattern as RPP over the course of the experiment: Resuscitation was able to maintain RBF (T1), esmolol reduced RBF (T2), and RBF recovered after stopping the esmolol infusion (T3) (Figure 1). In contrast, RVR was not significantly altered over the course of the experiment (Figure 3b and Table 1).

**Table 1:** Macrohemodynamic parameters and renal autoregulatory parameters per study phase. Data are expressed as mean ± *SD*. Renal perfusion pressure (RPP), Renal blood flow (RBF), renal vascular resistance (RVR), heart rate (HR) renovascular reactivity index (RVx), low frequency (LF), high frequency (HF), and very high frequency (VHF)

Variable	Baseline (T0) (*n* = 8)	Resuscitation (T1) (*n* = 8)	Esmolol (T2) (*n* = 8)	Stop (T3) (*n* = 8)	T0 versus T1^a^	T1 versus T2^a^	T2 versus T3^a^	T0‐T3^b^
Macrocirculation								
RPP (mmHg)	50 ± 7	47 ± 11	29 ± 10	40 ± 10	NS	***	**	***
RBF (ml/min)	137 ± 96	132 ± 96	81 ± 76	114 ± 79	NS	**	NS	***
RVR (mmHg/L/min)	51 ± 10	52 ± 11	46 ± 12	57 ± 11	NS	NS	NS	NS
HR (bpm)	124 ± 23	158 ± 12	100 ± 8	132 ± 11	***	***	***	***
Urea (mmol/L)	7.1 ± 1.3	7.5 ± 1.5	8.2 ± 2.3	7.5 ± 1.2	NS	NS	NS	NS
Autoregulation								
RVx (unit)	0.21 ± 0.3	0.56 ± 0.5	0.36 ± 0.5	0.7 ± 0.4	*	NS	*	*
Gain (ml/s/mmHg)—LF	0.9 ± 1.4	1.5 ± 1.9	2.3 ± 2.7	2.2 ± 2.1	NS	NS	NS	NS
Gain (ml/s/mmHg)—HF	1.1 ± 1.6	1.5 ± 1.9	2.2 ± 2.8	2.1 ± 2	NS	NS	NS	NS
Gain (ml/s/mmHg)—VHF	2.9 ± 3.1	2.6 ± 2	3.2 ± 3.1	4.1 ± 1.9	NS	NS	NS	NS
Phase (radians)—LF	0.1 ± 0	0.1 ± 0	0.1 ± 0	0.1 ± 0	NS	NS	NS	NS
Phase (radians)—HF	0.3 ± 0	0.3 ± 0	0.3 ± 0.2	0.3 ± 0.1	NS	NS	NS	NS
Phase (radians)—VHF	8 ± 0.6	7.6 ± 0.6	8 ± 0.6	7.8 ± 0.6	NS	NS	NS	NS
Coherence (unit)—LF	0.8 ± 0.1	0.9 ± 0.1	0.8 ± 0.1	0.9 ± 0.1	NS	NS	NS	**
Coherence (unit)—HF	0.8 ± 0.2	0.8 ± 0.2	0.9 ± 0.1	0.9 ± 0.1	NS	NS	NS	*
Coherence (unit)—VHF	0.8 ± 0.1	0.8 ± 0.1	0.8 ± 0.1	0.9 ± 0.1	NS	NS	NS	NS

NS, not significant.

a Significant changes between experimental phase (Paired Student's *t*‐tests).

b Repeated measures ANOVA was used to test for significance influence of the studied phases. *p < .05, **p < .01, ***p < .001.

### 3.3 Autoregulation

#### 3.3.1 Static time domain

**Figure 4:**
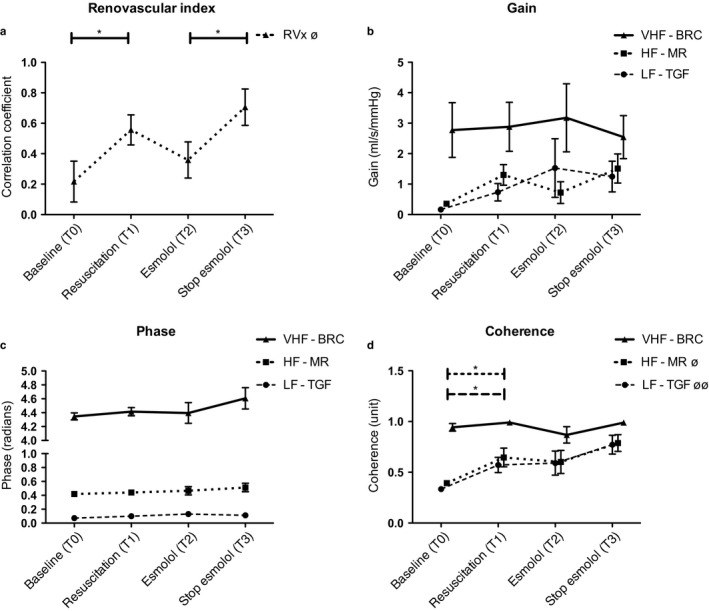
Renal autoregulation analyzed in the time domain, Renovascular reactivity index (RVx) (a) and using transfer function analysis in the frequency domain; (b) gain, (c) phase, and (d) coherence. Lf, Low frequency; TGF, tubuloglomerular feedback; HF, high frequency; MR, myogenic response; VHF, very high frequency; BRF, Baroreflex component. Repeated measures ANOVA was performed for each value and each frequency band, ^∅^ *p* < .05 and ^∅∅^ *p* < .01. Paired Student's *t*‐test was used to perform pairwise comparisons between phases. *p <.05

The RVx increased gradually over the course of the experiment (Figure 4a and Table 1), and showed a significant increase from baseline (0.21 ± 0.36) to resuscitation (0.56 ± 0.26). Showing an increased linear relationship between RPP and RBF during endotoxemia compared to baseline.

#### 3.3.2 Static autoregulation curves

**Figure 5:**
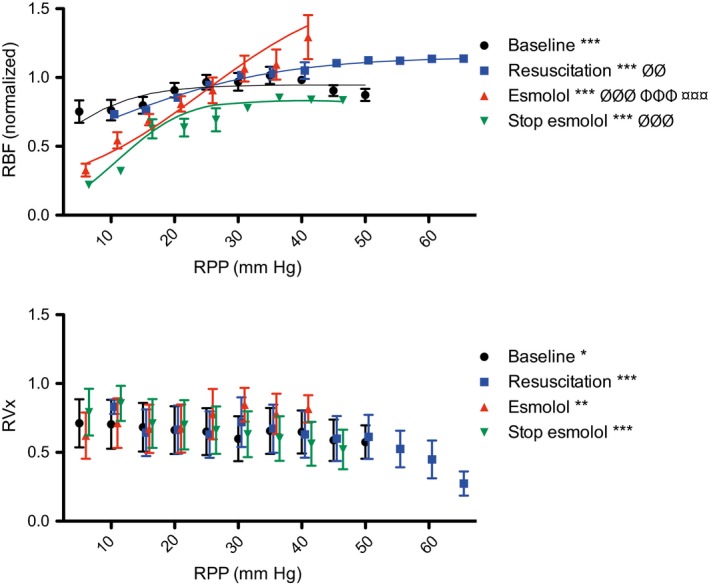
Renal autoregulation plots. (a) Renal blood flow (RBF) values are shown versus the simultaneous renal perfusion pressure (RPP). Lower limit of autoregulation (dashed lines) are shown per study phase; T0 at 0.3, T1 at 0.6, T2 at 1.2, and T3 at 0.5. (b) Renovascular reactivity index (RVx) values versus the simultaneous RPP (normalized to baseline). Data are expressed as mean and *SEM*. *p < .05, **p < .01, ***p < .001 influence of RPP on RBF or RVx (repeated measures one‐way ANOVA). ^∅∅^ *p* < .01 and ^∅∅∅^ *p* < .001 influence of RPP on RBF or RVx versus baseline (repeated measures two‐way ANOVA, time*treatment interaction term). 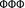: *p* < .001 influence of RPP on RBF during Esmolol versus resuscitation (repeated measures two‐way ANOVA, time*treatment interaction term). 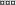: *p* < .001 influence of RPP on RBF during Esmolol versus Stop (repeated measures two‐way ANOVA, time*treatment interaction term)

Per study phase, both the static autoregulation curves (RPP vs. RBF) with their lower limit of autoregulation (Figure 5a and Table 1) and the dynamic autoregulation curves (RPP vs. RVx) are shown for comparison (Figure 5b and Table 1). During all four studied phases, RPP had a significant influence on both RBF and RVx. Furthermore, this relationship between RPP and RBF was significantly different during each phase (Figure 5a). During the baseline period, both RPP and RBF recovered from the surgical instrumentation. A further increase in RPP (above 70% of its baseline) did not cause a continued rise in RBF, except during esmolol infusion (T2) (Figure 5a). This resulted in the disappearance of the physiological “plateau” as this plateau reappeared after cessation of esmolol infusion, showing the reversibility of these effects. This is further emphasized by the lower limits of autoregulation. Under baseline conditions, the lower limit was at 30% of RPP. During infusion of esmolol, the lower limit increased to 120% and returned to 50% when esmolol infusion was stopped (Figure 5a). RVx showed large standard deviation when plotted against simultaneously recorded RPP values (Figure 5b). Fitting sigmoid curves to these data resulted in r‐square values as low as 0.2 (therefore not shown). Nevertheless, mean RVx increased with reductions in RBF, indicating pressure passivity with unregulated flow.

#### 3.3.3 Dynamic frequency domain

An example of a power spectra of the RPP and RBF signal during baseline, with the ranges of the different autoregulatory mechanisms is shown (Figure 2). Periodic events can be distinguished in both signals, notably heart rate (around 2 Hz) and respiration rate (around 0.4 Hz), and its harmonics appear as individual peaks in the power spectrum.

Using these power spectra for TFA showed that the gain and phase were not significantly influenced throughout the experiment in any of the autoregulatory frequency bands (Figure 4b and c). Similar to RVx, coherence, also as a measure for linearity, showed an increase over the course of the experiment for both the MR and TGF mechanism (Figure 4d).

## 4 DISCUSSION

### 4.1 Main findings

In the present experimental animal model, we found that RBF was preserved during resuscitated septic shock, but was strongly reduced as a result of esmolol infusion. This reduction was reversible and could be contributed to a reduction in RPP.

### 4.2 Renal blood flow

Renal blood flow in sepsis has been a long subject of debate given that approximately two thirds of experimental studies show a decreased RBF while in one third (Langenberg et al., 2005) the RBF was unchanged or even increased. These conflicting results appear to be affected by factors other than the induction of sepsis itself, including the consciousness of the animal, the recovery time after surgery, the hemodynamic pattern, and the (non‐)use of resuscitation maneuvers (Langenberg et al., 2005). We observed both phenomena; an initial decrease in RBF after LPS infusion and a full recovery by the resuscitation maneuvers (i.e., fluid and vasopressor therapy). Subsequently, esmolol infusion had a significant negative effect on RBF (accompanied with a trend towards RVR reduction), which was eliminated by stopping the esmolol infusion. This suggests that the reduced RBF was caused by the effects of esmolol itself and not by progressive endotoxemia. These acute negative effects of β‐blockers have been confirmed by others in both septic (Calzavacca et al., 2014) and non‐septic setting (Wilkinson, 1982).

### 4.3 Renal autoregulation

The large fluctuations in RBF within the experimental phases are clinically undesirable, but allowed us to study renal autoregulation by constructing autoregulation curves per phase. These curves revealed a pronounced protective mechanisms of renal autoregulation at (transient) excessive RPP values (Arendshorst, 1979; Arendshorst & Finn, 1977; Rhee et al., 2012). Protecting the kidney from high blood pressures is indeed the primary function of renal autoregulation in physiological circumstances (Bidani, Polichnowski, Loutzenhiser, & Griffin, 2013). The direct effect of esmolol on renal autoregulation is unknown. Theoretically, esmolol (a selective β1‐blockers) does not act directly on vascular function since β1‐receptors are not expressed in the renal vessels. However, some data suggest that β‐blockers might positively influence vasoactive mechanisms in acute endotoxemia (Du, Liu, Long, & Wang, 2017). A protective renal mechanism from hypertension during β‐blocker usage seems however irrelevant. We showed that the initial (T0 to T1) increased linearity between RBF and RPP was sustained during esmolol infusion. However, the unaltered dynamic autoregulation parameters indicate that this increased pressure dependency—impaired autoregulation can be attributed to a reduced RPP. The observation that acute septic shock did not damage the triple‐layered system (i.e., TGF, MR, and BRC) is consistent with literature (Burban et al., 2013). Aside from the septic shock and esmolol infusion, this transition in blood flow from autoregulated to pressure‐dependent would always be seen at those low RPP values (in our experiment below 25 mmHg). Here, the renal autoregulation is operating at the extremes of the physiological autoregulation curve, below the lower limit of renal autoregulation (Turkstra et al., 2000).

Both the autoregulation curves and time domain‐related RVx values refer to the relationship between average RPP and average RBF under steady‐state conditions, “dynamic” autoregulation parameters (i.e., gain, coherence, and phase) are of additional values by discriminating the different components of renal autoregulation (i.e., TGF, MR, and BRC). In our experiment, the coherence increased over time and was above 0.5 in all three bands after LPS infusion. The low spontaneous variability in blood pressure during baseline measurements could explain the low coherence between RPP and RBF in the low‐frequency range. This low coherence increases the chance of bias when interpreting the renal autoregulation during this phase (Brule, Kaam, Hoeven, Claassen, & Hoedemaekers, 2018). However, we showed relatively high coherence values in the low‐frequency band during endotoxic shock (T1 to T3). Admittedly less, but still expressing linearity of the somewhat nonlinear behaving TGF mechanism (Yip & Holstein‐Rathlou, 1996).

### 4.4 Cause of reduced RBF

We attributed the impaired autoregulation during esmolol infusion to a reduced RPP rather than to damaged autoregulation mechanisms. These perfusion deficits indeed play an important role in the development of renal dysfunction in sepsis (Post, Su, et al., 2017). Herein, this deficit is largely explained by a markedly increased CVP, resulting in a significantly reduced RPP and RBF (Pearson correlation *p* < .05). This venous congestion disappeared rapidly after stopping esmolol infusion (also shown in the related paper (Loon et al., 2018)). While it is unknown to which extend the driving pressure and backpressure are actually experienced by the kidney, it is undisputed that an increased CVP is associated with impaired renal function and independently related to all‐cause mortality in a broad spectrum of patients with cardiovascular disease (Damman et al., 2009). The renal autoregulatory mechanisms might be better in maintaining RBF with a drop in ABP compared to an increase in CVP. This concept of increased CVP, being transmitted to the renal veins and kidneys leading to renal dysfunction is supported by a substantial amount of literature as early as in the 1930s (Mullens et al., 2009). A retrospective study of septic patients in the ICU showed associations between a higher CVP and acute kidney injury, but not between MAP or cardiac output and acute kidney injury (Legrand et al., 2013).

### 4.5 Clinical implications

In the acute septic setting, autoregulation is preserved but impaired by esmolol. However, clinical diligence and caution are necessary when treating septic shock with esmolol in the acute phase since esmolol reduced RPP to critical values thereby significantly reducing RBF. In addition, we showed the importance of CVP in regulating RBF. Although controversial and in contrast to others (Marik & Cavallazzi, 2013), we showed that assessment of CVP can provide clinical information when guiding intravenous fluids administration considering perfusion of the kidneys.

In light of our results, future research should focus on studying the impact of the backpressure on RBF and improving noninvasive techniques for assessing RBF during the treatment of sepsis patients. Contrast‐enhanced ultrasound could be such a safe and noninvasive imaging technique for assessment of tissue blood flow, although its accuracy has been questioned in recent experiments (Cokkinos et al., 2013). Furthermore, with RBF determining oxygen delivery and sodium absorption being the main contributor to oxygen consumption, studying the effect both may help in preventing esmolol induced renal hypoxia.

### 4.6 Limitations

Knowing that the ideal model of sepsis does not exist, our model has proven to be highly controlled, reproducible, and representative for several hallmarks of sepsis (Fink, 2014).

In order to monitor urine production by the urethral catheterization we were limited to using only female animals. Note, the relatively young lambs had considerable lower perfusion pressure compared to their adult counterparts in other literature (Fan, Mukaddam‐Daher, Gutkowska, Nuwayhid, & Quillen, 1994). Although clinically irrelevant, the short period between LPS infusion and the start of the resuscitation maneuvers did not allow for autoregulation analysis of that period. Furthermore, we only assessed the acute septic setting. Burban et al. (2013) confirmed the preservation of the renal autoregulation within the first hours of sepsis. Indeed, several experimental data have shown that renal hemodynamics may be different at the initiation, maintenance, and recovery phases of septic AKI (Benes et al., 2011; Langenberg, Wan, Egi, May, & Bellomo, 2006). The use of the miniature probes to measure RBF require some special consideration. Special care was taken to avoid positions that may have temporarily obstructed flow. Optimal positioning of the probe is not trivial and is critical to determine the maximal flow through the renal artery (Welch, Deng, Snellen, & Wilcox, 1995). We did not use biomarkers from blood samples to assess kidney function, and failed to quantify urine production and glomerular filtration rate (GFR). The relative low urine production made it impossible to correlate diuresis to one of the studied phases. GFR would have helped to separate the effect of afferent and efferent arterioles on RBF.

All renal autoregulation assessment methods have their limitation; First, both RVR and RVx were measured in the time domain, making them relatively blunt metrics that treat the separate autoregulatory elements in the kidney as a single unit, detecting overall pressure passivity, but no failure of an individual component of reactivity and they do not take the direction of change into account (Rhee et al., 2012). Second, the FTA assumes linearity between the input and output signal, but the renal autoregulatory system usually displays some degree of nonlinear behavior (Yip & Holstein‐Rathlou, 1996). FTA assumes the properties of the system are stationary or have constant mean and variance over time; however, this assumption may not always be valid, particularly when prolonged time series are used in hemodynamically unstable subjects (Chon, Zhong, Moore, Holstein‐Rathlou, & Cupples, 2008). Finally, the averaged autoregulation curves presented in the Figure 5 underestimate the power of autoregulation of each individual animal, as not all lower limits of autoregulation are exactly similar (Turkstra et al., 2000). Individual curves, however, bared a too small range in RPP values to calculate these limits per study phase. Despites the limitations of these separate renal autoregulation assessment methods, together they provide a comprehensive view of the kidney's capabilities of managing RBF. Therefore, depending on specific research purposes, the choice, and interpretation for renal autoregulation assessment should be weighed carefully.

## 5 CONCLUSION

Using an acute endotoxic septic shock sheep model, we showed that renal autoregulation remains unaffected in situations of resuscitated septic shock, but concurrent esmolol infusion significantly increased the pressure dependency of RBF.

## CONFLICT OF INTEREST

None declared.

## AUTHOR CONTRIBUTIONS

L.M.v.L conceptualized and designed the study, acquired the data, analyzed and interpreted the data, drafted the manuscript, and critically revised the manuscript. G.A.R analyzed and interpreted the data, and critically revised the manuscript. J.G.v.H. conceptualized and designed the study, analyzed and interpreted the data, and critically revised the manuscript. P.H.V.: conceptualized and designed the study, and critically revised the manuscript. J.L.: conceptualized and designed the study, acquired the data, analyzed and interpreted the data, and critically revised the manuscript.

## ETHICS APPROVAL AND CONSENT TO PARTICIPATE

This experiment was performed after approval by the local ethics committee on animal research of the Radboud University Nijmegen Medical Center (RUNMC License number RU‐DEC 2014–10) and in full compliance with Dutch and European legal requirements on the use and protection of laboratory animals.

## CONSENT FOR PUBLICATION

Not applicable.

## AVAILABILITY OF DATA AND MATERIAL

The materials described in this manuscript, including all relevant raw data, is freely available to any scientist wishing to use them for noncommercial purposes.
